# Link prediction of heterogeneous complex networks based on an improved embedding learning algorithm

**DOI:** 10.1371/journal.pone.0315507

**Published:** 2025-01-07

**Authors:** Lang Chai, Rui Huang

**Affiliations:** 1 School of Mathematics and Statistics, Chongqing Jiaotong Univeristy, Chongqing, China; 2 School of Foundation Courses, Chongqing Institute of Engineering, Chongqing, China; Universidad Rey Juan Carlos, SPAIN

## Abstract

Link prediction in heterogeneous networks is an active research topic in the field of complex network science. Recognizing the limitations of existing methods, which often overlook the varying contributions of different local structures within these networks, this study introduces a novel algorithm named SW-Metapath2vec. This algorithm enhances the embedding learning process by assigning weights to meta-path traces generated through random walks and translates the potential connections between nodes into the cosine similarity of embedded vectors. The study was conducted using multiple real-world and synthetic datasets to validate the proposed algorithm’s performance. The results indicate that SW-Metapath2vec significantly outperforms benchmark algorithms. Notably, the algorithm maintains high predictive performance even when a substantial proportion of network nodes are removed, demonstrating its resilience and potential for practical application in analyzing large-scale heterogeneous networks. These findings contribute to the advancement of link prediction techniques and offer valuable insights and tools for related research areas.

## Introduction

Nowadays, the interactions between various entities are becoming increasingly complex and frequent, leading to the generation of massive amounts of unstructured data. In the field of complex network science, scholars often abstract this unstructured data into complex networks composed of nodes and edges [1]. By studying these complex networks, researchers can gain significant insights into solving real-world problems [2]. Among these studies, link prediction in complex networks is a crucial problem in the science of complexity [3, 4]. Link prediction aims to forecast unobserved or potential future edges in a complex network by analyzing the observable nodes and edges [5, 6]. Theoretically, link prediction has the potential to reveal the mechanisms underlying the generation of complex network structures [7, 8]. Practically, it has wide applications across various fields, such as extracting hidden information in military combat networks of weapon systems [9]; detecting fraud in the financial sector [10]; predicting connections between neurons in E.coli and chemical reactions among components in metabolic networks in biology [11]; providing more precise product recommendations in consumer networks based on user-product relationships [12]; inferring associations between small molecules with unclear chemical information and proteins in drug discovery [13]; and identifying potential suspects in criminal investigations through social network analysis to swiftly dismantle criminal organizations [14].

In the filed of link prediction, numerous outstanding works have advanced its development. Clauset et al. proposed a hierarchical model-based approach to link prediction, highlighting the significance of hierarchical organization in networks [15]. Guimerà and Sales-Pardo further investigated the impact of missing and spurious interactions on network reconstruction [16]. Matrix factorization has been extensively studied in the domain of link prediction, providing robust and interpretable frameworks for uncovering hidden relationships in networks [17–19]. Moreover, several link prediction algorithms based on hybrid methods have been extensively studied [20–22]. Lu et al. explored the predictability of link prediction in complex networks and introduced a framework for assessing the predictability of network structures [23]. Furthermore, these studies [24–26] have explored link predictability from various perspectives, yielding numerous valuable insights and results.

However, most of the link prediction researches for complex networks mainly focuses on homogeneous networks currently [27–29]. In the real world, From the perspective of node types and edge types [30], complex networks can be classified into homogeneous and heterogeneous networks. Heterogeneous complex networks have multiple types of node types, which are more advantageous in characterizing complex real-world systems [31]. And Heterogeneous networks are increasingly prevalent, facilitating communication among different types of individuals. Examples of such networks include recommendation system [32], where recommendation links exist between users and commodities; scientific citation network [33], where the citation links connect scholars and articles, and protein synthesis networks [34], which exist different types of links between various amino acids. Unfortunately, the research on heterogeneous complex network links prediction is still relatively weak, but its importance has become increasingly prominent in both theoretical and practical terms.

Thanks to the swift progress in deep learning methods, as seen in references [35–37], researchers have effectively combined deep learning with link prediction in heterogeneous networks, leading to a series of research successes. A significant milestone was the introduction of meta-paths to capture the nuances of heterogeneous networks [38]. These meta-paths are effective at representing the semantic content and structural connections within the networks. Since their inception, meta-paths have become a leading approach in link prediction for heterogeneous networks. For example, using meta-paths, Shakibian and colleagues [39] proposed a similarity index for predicting links in heterogeneous networks by analyzing co-occurrence matrices. Additionally, Shakibian introduced an unsupervised learning algorithm based on a multi-core single-class SVM, leveraging meta-paths [40]. However, while these meta-path-based algorithms characterize network information, they do not fully utilize deep learning’s strengths in capturing network structure.

The goal is to combine the benefits of meta-paths with deep learning to create advanced algorithms for predicting links in heterogeneous networks. In 2017, Dong and team [41] presented the Metapath2vec framework, which integrates meta-paths with the word2vec technique. This framework sorts the types of neighboring nodes and maps similar types into the same potential space. To better capture the structural information in heterogeneous networks, researchers have started to include node weights and meta-path weights in the embedding process. In 2019, Phu Pham and others [42] proposed the W-MetaPath2Vec algorithm, building on Metapath2vec, assigning weights to nodes based on topic relevance and training the model with these weighted nodes. In 2020, Zhang and colleagues [43] developed a method that assigns weights to each meta-path and trains the embedding model following the Metapath2vec approach.

These achievements have significantly pushed forward the study of link prediction in heterogeneous networks. However, current research has not yet fully considered how different local structures in these networks contribute to embedding learning. Just as the graph attention network GAT [44] acknowledges varying contributions from local structures to node feature learning, incorporating this local structural information into embedding learning for heterogeneous networks is an issue that needs further exploration.

Following the analysis presented, this paper introduces an innovative method for link prediction in heterogeneous networks. Our method integrates network embedding with local structural insights, allowing us to allocate weights to various local structures within heterogeneous networks. This facilitates the creation of a new objective function for embedding learning and the development of a unique embedding learning algorithm for heterogeneous networks, namely the Structural Weighted Metapath2vec (SW-Metapath2vec) approach. The SW-Metapath2vec algorithm initiates with random walks to produce meta-path sequences. Subsequently, it applies a structure-weighted strategy for embedding learning in heterogeneous networks. We then utilize the resulting node embedding vectors to forecast links within these complex networks. To substantiate the efficacy of our proposed algorithm, we perform experiments on both real-world and synthetic datasets, contrasting our outcomes with those of benchmark algorithms.

In summary, the proposed SW-Metapath2vec algorithm represents a promising approach for link prediction in heterogeneous networks. By fusing network embedding with local structural information and designing a novel embedding learning algorithm, we provide a powerful method for analyzing and predicting links in complex networks.

## Preliminaries

To better introduce the main content of this paper, this section provides an overview of the essential preparatory knowledge on heterogeneous networks, meta-paths, and the cosine similarity index.

### Heterogeneous network

**Difinition 1 (Heterogeneous network)** On the network *G* = (*V*, *E*, *T*_*V*_, *T*_*E*_), where *V*, *E* are the node and edge set, *T*_*V*_, *T*_*E*_ are the node type and edge type collections, respectively. Then there exists *π*: *V* → *T*_*V*_ and Ψ: *E* → *T*_*E*_, making the node *v* ∈ *V* and the link *e* ∈ *E* among *G* have *π*(*v*)∈*T*_*V*_, Ψ(*e*)∈*T*_*E*_, and |*T*_*V*_| ≥ 2, then the network *G* is called heterogeneous network.

**Definition 2 (Network scheme)** Network *G*_*T*_(*T*_*V*_, *T*_*E*_) is a network scheme if the heterogeneous network *G*(*V*, *E*, *T*_*V*_, *T*_*E*_) is a directed network under the node map *π*: *V* → *T*_*V*_ and the link map Ψ: *E* → *T*_*E*_.

### Meta-path

**Definition 3 (Meta-path)**: Meta-path P is a trace defined on the network scheme *G*_*T*_(*T*_*V*_, *T*_*E*_), and the mode is $T_{V}^{1}\overset{T_{E}^{1}}{\to }T_{V}^{2}\overset{T_{E}^{2}}{\to }\cdots \overset{T_{E}^{l-1}}{T_{V}^{l}}$, where $T_{V}^{i}\in T_{V},i=1,2,\cdots \left|T_{V}\right|$, $T_{E}^{j}\in T_{E},j=1,2,\cdots ,\left|T_{E}\right|$.

**Remark 1**: Meta-path P fixes the link combination $T_{E}^{\prime }=T_{E}^{i_{1}}\circ T_{E}^{i_{2}}\circ \cdots \circ T_{E}^{i_{l-1}}$ between node type $T_{V}^{1}$ and node type $T_{V}^{l}$, where “∘” is the links combination operator.

**Fig 1:**
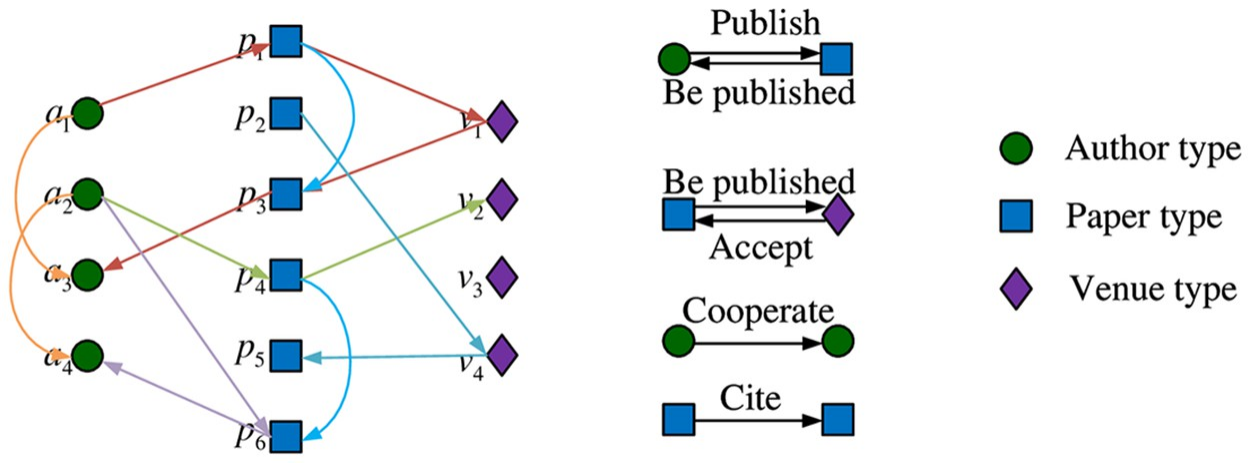
Meta-paths of the “Author-Paper-Venue” network.

To illustrate the meta-path, we take a simple “Author-Paper-Venue” network as an example. Fig 1 shows some meta-paths in the network. We call the path *p*_1_: *a*_1_ → *p*_1_ → *v*_1_ → *p*_3_ → *a*_3_ a meta-path trace that follows the meta-path $P_{1}:APVPA$, where the “A” represents the “Author”, the “P” represents the “Paper”, and the “V” represents the “Venue”. The semantic relationships of this case show that both the author *a*_1_ and the author *a*_3_ have published their papers in the journal *v*_1_, so the possibility of citation or cooperation between the two authors is greater. In parallel, the path *p*_2_: *a*_2_ → *p*_4_ → *v*_*v*_ is a meta-path trace following the meta-path $P_{2}:APV$. In particular, (1) there is often more than one meta-path trace following a meta-path P. For example, *p*_3_: *a*_1_ → *p*_1_ → *v*_1_ is also a meta-path trace following $P_{2}$. (2) The length of the required meta-path trace can be specified for each application.

There have been numerous advancements in the generation of meta-path traces [45–48]. One common method is using a random walker to generate these traces. The following provides a brief introduction to the process of obtaining a meta-path using this approach.

When using a random walker to generate a meta-path trace, it is necessary to randomize the starting nodes of the random walker R. Different meta-path traces can be obtained by adjusting the hyper-parameters such as the walk length *L* and the times of node traversal *N*. Given *G*(*V*, *E*, *T*_*V*_, *T*_*E*_) and the meta-path $P:T_{V}^{1}\overset{T_{E}^{1}}{\to }T_{V}^{2}\overset{T_{E}^{2}}{\to }\cdots \overset{T_{E}^{l-1}}{\to }T_{V}^{l}$, the meta-path trace can be generated as follows.

Firstly, randomly set the $T_{V}^{1}$ type start node $v_{T_{V}^{1}}^{0}$ in the meta-path trace. Then, according to the transition probability of the random walker R from step *i* to step *i* + 1 under the meta-path P

$$\begin{array}{c} p_{R}\left(v_{t+1}^{i+1}|v_{t}^{i},P\right)=\{\begin{array}{cc} \frac{1}{|N_{t+1}\left(v_{t}^{i}\right)|}, & \left(v_{t}^{i},v_{t+1}^{i+1}\right)\in E\&\pi \left(v_{t+1}^{i+1}\right)=t+1\\ 0, & other \end{array} \end{array}$$ (1)

generate the $T_{V}^{2}$ type node $v_{T_{V}^{2}}^{1}$. The remaining are deduced by looping through the process until we get a meta-path trace of the specified length. In Eq (1), $v_{t}^{i}$ is the *t* type node in step *i*, and $|N_{t+1}\left(v_{t}^{i}\right)|$ represents the number of *t* + 1 type neighbor nodes of type *t* nodes in step *i*.

**Remark 2**. Generally, using an symmetric meta-path is more conducive to capturing effective information, especially the similarity between nodes; an asymmetric meta-path has a very limited ability to obtain useful information. For example, the meta-path *APVPA* in Fig 1 can reflect the cooperation between two authors and the possibility of article quotation; the meta-path *APV* can only show that an author publishes an article in a journal.

## Materials and methods

### Embedding learning with structure weights for general networks

The embedding learning model of complex networks was inspired by the word embedding methods in natural language processing (NLP) [49–51]. It learns the embedding vector of each node in the network. For a general complex network *G* = (*V*, *E*), a traditional embedding learning objective function was defined as

$$\begin{array}{c} \underset{g}{\text{arg}\;\text{max}}\prod_{v\in V}\prod_{c\in N(v)}p\left(c|v\right), \end{array}$$ (2)

where the embedding function $g:V\to R^{d}$ maps each node *v* ∈ *V* into a *d* dimensional vector space [0, 1]^*d*^, *d* = |*V*|. *N*(*v*) is the neighbor set of the node *v* in the network *G*. The conditional probability *p*(*c*|*v*) represents the probability that the node *c* is a neighbor node of node *v*. It also can describe the local structure of the network. *p*(*c*|*v*) is defined as

$$\begin{array}{c} p\left(c|v\right)=\frac{e^{X_{c}\cdot X_{v}}}{\sum_{u\in V}e^{X_{c}\cdot X_{u}}},c,v,u\in V, \end{array}$$ (3)

where *X*_node_ = *g*(node), node = *c*, *v*, *u* represents the potential embedding, respectively. It should be noted that Eq (2) only considers the likelihood probability of node local structure. There is no deeper level structural information among nodes *c*, *v* and *u*.

**Definition 4**. The matrix ***X*** is called the embedding matrix, in which the row vectors correspond to the embedding vector of each node. For example, *X*_*c*_, *X*_*v*_, and *X*_*u*_ are in the *c*, *v*, and *u* row of the matrix ***X***, respectively.

**Fig 2:**
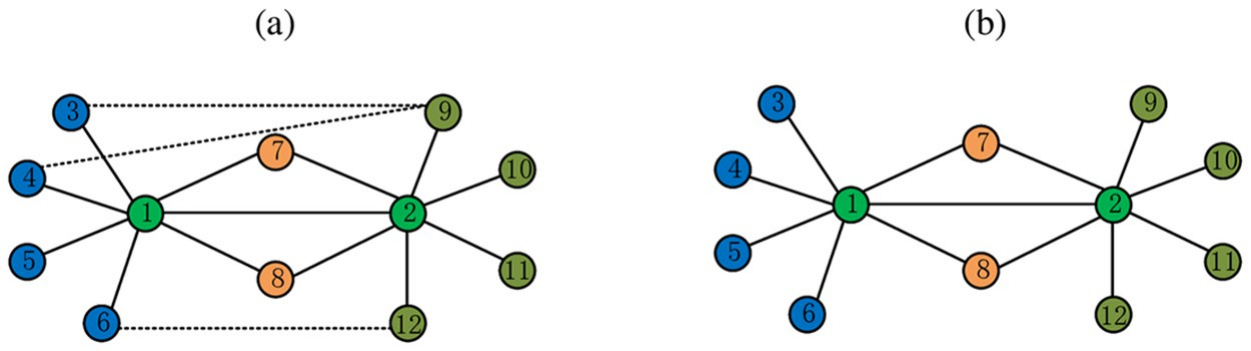
The local structure of the link between node “1” and node “2”.

In this paper, we consider assigning different weights to various local structures in heterogeneous networks to measure their contributions to network embedding. As shown in Fig 2, when embedding the node “1” and the node “2”, the link strength between the nodes “1” and “2” in Fig 2(a) is stronger than in Fig 2(b) in terms of network semantics. This is because, in Fig 2(a), there are some links between the neighbor nodes of the node “1” and the node “2”. These links between the neighbor nodes enhance the strength of the link between node “1” and node “2” and may indicate that node “1” and node “2” are more likely to belong to the same level in the network. In contrast, in Fig 2(b), there are no links between the neighbor nodes of node “1” and node “2”, suggesting that the link between node “1” and node “2” may be serendipitous, and the likelihood that they belong to the same level is relatively low. Based on this idea, we define the following formula to determine the weight for the local structure in networks.

$$\begin{array}{c} \Gamma \left(v,c\right)=\frac{1+\#(N(v),N(c))}{\left|N(v)|\cdot |N(c)\right|}, \end{array}$$ (4)

where *N*(*v*) and *N*(*c*) are the neighbors set for the node *v* and *c* separately, #(*N*(*v*), *N*(*c*)) represents the number of links between the nodes of *N*(*v*) and *N*(*c*). Specially, we regard there exists a link between the nodes of *N*(*v*) and *N*(*c*) if a node *m* is the common neighbor node between nodes *v* and *c*, such as the node “7” and “8” in Fig 2(a). Therefore, in Fig 2, the $\Gamma \left(1,2\right)=\frac{6}{49}$ and $\Gamma \left(1,2\right)=\frac{3}{49}$, respectively.

Combined with the Eq (2) and the formula Eq (4), in this paper we define the embedding learning with structure weight as follows

$$\begin{array}{c} \underset{g}{\text{arg}\;\text{max}}\prod_{v\in V}\prod_{c\in N(v)}\Gamma \left(v,c\right)\cdot p\left(c|v\right). \end{array}$$ (5)

In the traditional embedding objective function (2), the contribution of each local structure of the network to the embedding is equal. While in object (5), the weight value of different local structures can highlight the structural impact in the link prediction. This means that the features obtained by the embedding function *g* are more suitable for link prediction.

### Embedding learning with structure weight for heterogeneous networks

For the heterogeneous networks, we also design a new embedding method as the Eq (6) in the following. Given a heterogeneous network *G*(*V*, *E*, *T*_*V*_, *T*_*E*_) and |*T*_*v*_| ≥ 2, for any node *v*_*t*_ ∈ *V*, The heterogeneous networks embedding learning with structure weight is defined as

$$\begin{array}{c} \underset{g}{\text{arg}\;\text{max}}\sum_{t,t^{\prime }\in V}\sum_{v_{t}\in V}\sum_{v_{t^{\prime }}\in N_{t^{\prime }}\left(v_{t}\right)}\text{log}\left[\Gamma \left(v_{t},v_{t^{\prime }}\right)\cdot p\left(v_{t^{\prime }}|v_{t}\right)\right], \end{array}$$ (6)

where *N*_*t*′_(*v*_*t*_) is the neighbor set of the node *v*_*t*_, which node type is *t*′. Γ(*v*_*t*_, *v*_*t*′_) is the weight between nodes *v*_*t*_ and *v*_*t*′_ according to Eq (4).

When the scale of the network is extremely huge, the optimization of the objective function (6) is relatively difficult. To overcome this obstacle, the negative sampling strategy and binary logistic regression are used to train the embedding model [52]. The negative sampling strategy replaces the global parameters update, which effectively improves the optimization efficiency.

For descriptive convenience, we denote a positive sample as (*v*_*t*_, *v*_0_) and suppose there are *K* negative samples (*v*_*t*_, *v*_1_), (*v*_*t*_, *v*_2_), ⋯, (*v*_*t*_, *v*_*K*_). Given binary logic regression, it is seen that positive and negative samples must meet

$$\begin{array}{c} p\left(y_{0}=1|\left(v_{t},v_{0}\right)\right)=\Gamma \left(v_{t},v_{0}\right)\cdot \sigma \left(X_{v_{t}}\cdot X_{v_{0}}\right), \end{array}$$ (7)

and

$$\begin{array}{c} p\left(y_{k}=0|\left(v_{t},v_{k}\right)\right)=1-\Gamma \left(v_{t},v_{0}\right)\cdot \sigma \left(X_{v_{t}}\cdot X_{v_{k}}\right),k=1,2,\cdots ,K. \end{array}$$ (8)

According to Eqs (7) and (8), we have

$$\begin{array}{c} L\left(X\right)=\text{log}\left(\Gamma \left(v_{t},v_{0}\right)\cdot \sigma \left(X_{v_{t}},X_{v_{0}}\right)\right)+\sum_{k=1}^{K}E_{v_{k}∼F\left(u\right)}\text{log}\left(1-\Gamma \left(v_{t},v_{0}\right)\cdot \sigma \left(X_{v_{t}}\cdot X_{v_{k}}\right)\right), \end{array}$$ (9)

where the $\sigma \left(x\right)=\frac{1}{1+e^{-x}}$. $F\left(u\right)=f{\left(u\right)}^{\frac{3}{4}}/\sum_{v\in V}f{\left(v\right)}^{\frac{3}{4}}$ is the probability distribution of negative samples, and *f*(*u*) is the frequency of node *u* in the network.

For Eq (9), the gradient is

$$\begin{array}{c} \left\{ \begin{array}{cc} & \frac{\partial L(X)}{\partial X_{v_{t}}}=\sum_{k=0}^{K}\left(\Gamma \left(v_{t},v_{k}\right)\cdot \Theta_{v_{k}}\left[v_{t}\right]-\Gamma \left(v_{t},v_{0}\right)\cdot \sigma \left(X_{v_{t}},X_{v_{k}}\right)\right)X_{v_{k}}\\ & \frac{\partial L(X)}{\partial X_{v_{k}}}=\left(\Gamma \left(v_{t},v_{k}\right)\cdot \Theta_{v_{k}}\left[v_{t}\right]-\Gamma \left(v_{t},v_{0}\right)\cdot \sigma \left(X_{v_{t}},X_{v_{k}}\right)\right)X_{v_{t}} \end{array},\right. \end{array}$$ (10)

where $\Theta_{v_{k}}\left[v_{t}\right]$ is the indicative function, which has the value 1 when *v*_*t*_, *v*_*k*_ is a positive sample, or 0 when (*v*_*t*_, *v*_*k*_) is a negative sample.

The potential vectors $X_{v_{t}},X_{v_{0}},X_{v_{1}},\cdots ,X_{v_{K}}$ can be updated by Eq (10) until the proper embedding vectors are obtained.

**Fig 3:**
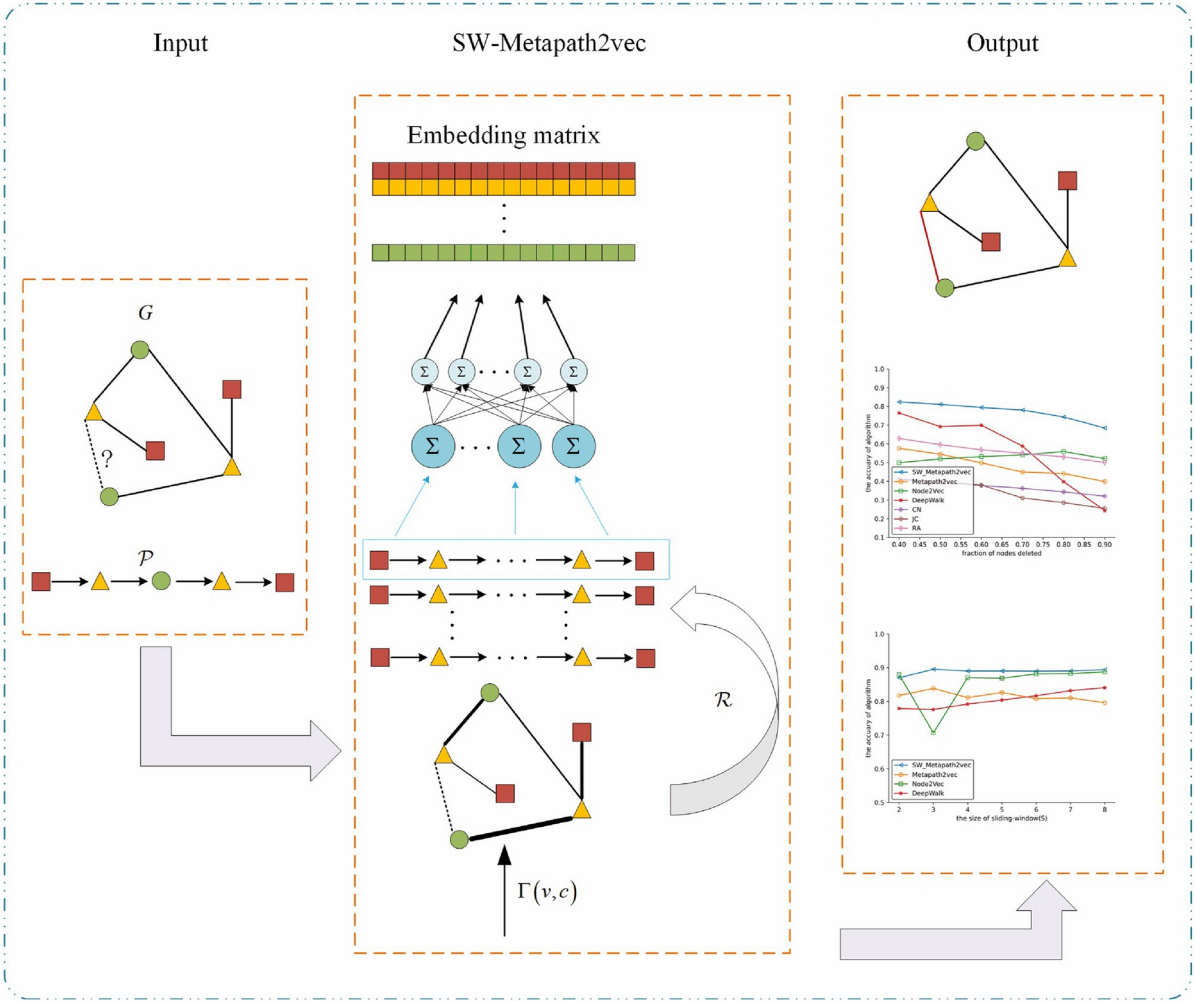
Framework of SW-Metapath2vec algorithm.

Based on the above analysis, Fig 3 describes the framework of the SW-Metapath2vec algorithm proposed in this paper. As shown in Fig 3, the first block “Input” is the data preparing stage. In this step, we should design the appropriate meta-path P in view of the object of link prediction. The second block is the main method “SW-Metapath2vec” algorithm, using Eq (4) to assign weight to different local structures, and then generate the meta-path traces according to P, finally through the objection function (6) obtain the embedding vector of each node. The final block “Output” includes the link prediction by embedding vectors and other analysis about the SW-Metapath2vec, such as hyper-parameters sensitivity, robustness. In addition, we also provide pseudo-code for the SW Metapath2vec algorithm. The available code can be found in the repository https://github.com/pinglanchu/SW-Metapath2vec.

**Algorithm 1**: SW-Metapath2vec link prediction algorithm

 **input** : Heterogeneous graph *G*, link type E, testing ratio *r*

 **output**: node embedding *X*, AUC, Precision

 // Division of training and testing sets

1 testing links $E^{T}=random.choose\left(G,E,r\right)$; Randomly select links of the specified link type E with a ratio of *r* from *G*;

2 training network *G*^*train*^ = *G*.*remove*_*links*(*E*^*T*^); Remove the selected testing links *E*^*T*^ from *G*;

3 positive testing network $G_{pos}^{test}=G_{Null}.copy\_node\left(G\right).add\left(E^{T}\right)$; Copy the nodes in network *G* to an empty network *G*_*Null*_, and then add *E*^*T*^ to the network to build a test network;

4 negative testing network $G_{neg}^{test}=random.choose\left(G^{\prime },E,r\right)$; Randomly select links of the specified link type E with a ratio of *r* from *G*′, *G*′ is a supplementary network of *G*; // Training SW-Metapath2ve

5 **for** *node v in G*^*train*^(*V*) **do**

6  Calculating local structure weight Γ(*v*, *c*) by Eq (4);

7  Training node embedding *X* by Eq (10);

8 **end**

  // Testing SW-Metapath2ve

9 **for** *link* = (*node*_*src*, *node*_*dst*) *in* $G_{pos}^{test}\left(E\right)$ **do**

10  sim = cosine(X[node_src], X[node_dst]);

11  real_label = 1;

12 **end**

13 **for** *link* = (*node*_*src*, *node*_*dst*) *in* $G_{neg}^{test}\left(E\right)$ **do**

14  sim = cosine(X[node_src], X[node_dst]);

15  real_label = 0;

16 **end**

17 AUC = auc(real_label, sim), Precision = precision(real_label, sim)

## Results

### Datasets

To verify the effectiveness and feasibility of the SW-Metapath2vec algorithm proposed in Section: Materials and methods. In this section, we will conduct some experiments with real and synthetic heterogeneous networks.

(1) **ACM** [53] contains various types of nodes, such as “papers”, “authors”, and “fields”, and multiple types of edges between them, all of which are from the ACM digital library. The ACM dataset we used contains 4025 papers (P), 7351 authors (A), and 72 fields (F). Also, it concludes two types of edges, which are the “paper-author”, and “paper-field”.
(2) **DBLP** [54] is a scientific literature dataset. We extract a subset of DBLP with 4057 authors (A), 6385 papers (P), 4108 terms (T), and 4128 venues (V). The DBLP heterogeneous network contains three types of edges, which are the “paper-author”, “paper-term”, “paper-venue”. In this paper, we used the basic information in the literature to construct heterogeneous connections between “paper-author”, “paper-term”, and “paper-venue”. The specific information is shown in Table 1.
(3) **Last.fm** [55] is music recommendation dataset. The Last.fm heterogeneous network contains 1892 users (U), 9524 artists (A), and 5612 tags (T); and includes three types of edges, which are the “user-user”, “user-artist”, and “artist-tag”.

**Table 1:** General information for the three real heterogeneous networks.

Dataset	Relations(A-B)	Number of A	Number of B	Number of A-B	Meta-paths
ACM	paper-author	4025	7351	5599	PAPFP
	paper-field	4025	72	4025	
DBLP	paper-author	6385	4057	6820	APTPVPA
	paper-term	6385	4057	6280	
	paper-venue	6385	4128	1235	
Last.fm	user-user	1892	1892	6023	UATAU
	user-artist	1892	9524	6023	
	artist-gag	9524	5612	4142	

To ensure the fairness and effectiveness of numerical experiments, heterogeneous complex networks are divided into training and testing sets based on the predicted edge type “etype” in each experiment. Among them, the nodes in the training set and the testing set are consistent. Given the proportion *r* of the testing set, remove the links of the “etype” from the original network, and the remaining heterogeneous complex network is the positive training set network; In addition, the network composed of non-links in the original network is taken as the negative network, and *r* proportion of “etype” type edges are removed from the negative network. The remaining heterogeneous complex network is the negative training set network. At this point, the removed “etype” type links constitute the positive and negative test set networks, respectively.

**Fig 4:**
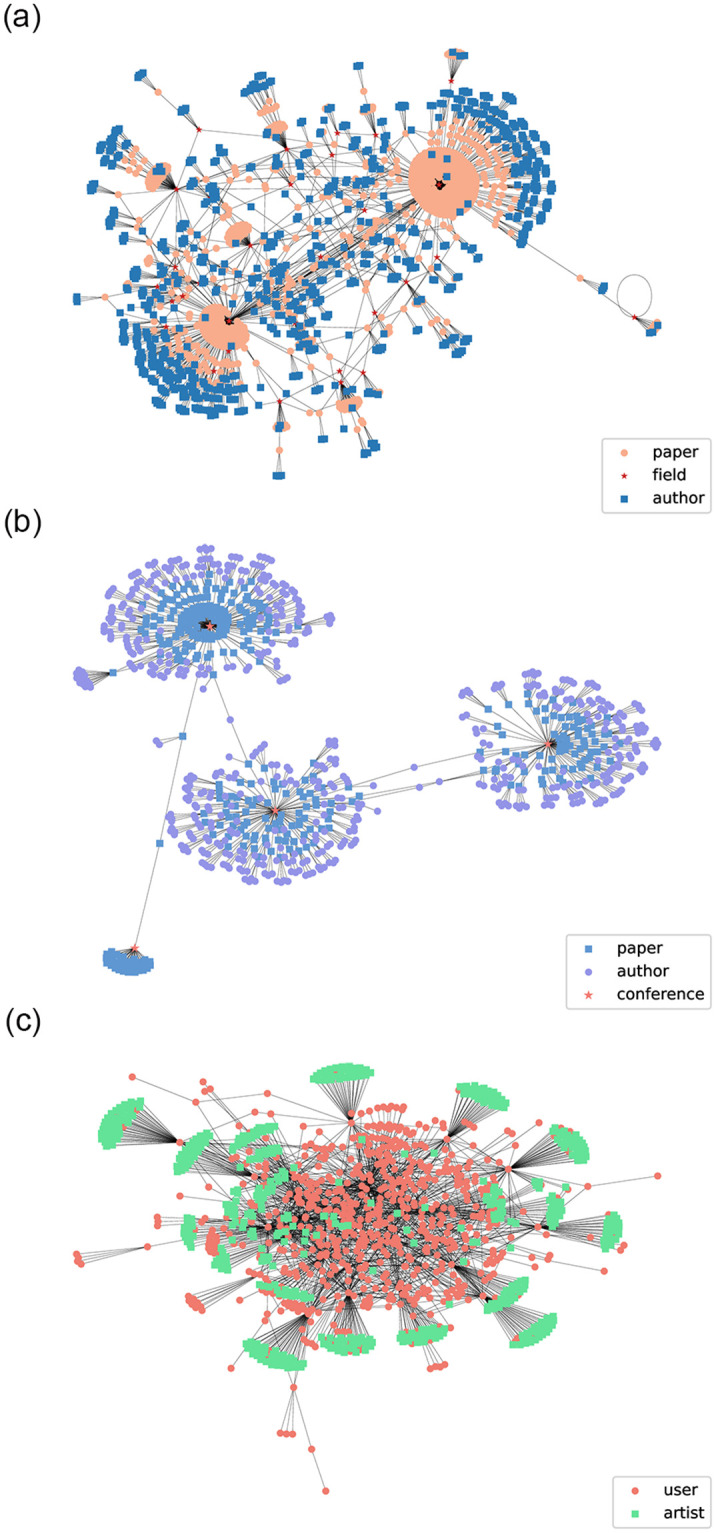
Three real heterogeneous complex networks. (a) ACM dataset network; (b) DBLP dataset network; (c) Last.fm dataset network.

Fig 4 displays the “paper-author-venue” network in the ACM dataset, the “author-paper-field” network in the DBLP dataset, and the “user-artist” network in Last.fm dataset. Then the Table 1 shows the basic information about the three datasets, where the Meta-paths are manually set in this paper.

In addition, we have generated two synthetic networks for the experiments, as seen in Table 2. |*V*| and |*E*| are the number of nodes and links respectively. < *k* > and < *d* > are average degree and average shortest path length. Finally, *ρ* denotes the density of the network and the #*cs* is the number of connected components.

**Table 2:** Topological properties of the two synthetic networks.

Synthetic networks	|*V*|	|*E*|	< *k* >	< *d* >	*ρ*	#*cs*
ER(2000, 0.03)	2000	59565	59.565	2.129	0.029	1
WS(2000, 10, 0.03)	2000	10000	10	6.906	0.005	1

### Baseline algorithms

To better compare the performance of the SW-Metapath2vec algorithm proposed in this paper with other classic link prediction algorithms for heterogeneous complex networks, we selected several representative link prediction algorithms as benchmark algorithms. The following is a brief introduction to these algorithms for comparison.

(1) **CN**: [56] The more common neighbors two nodes have, the higher the similarity between them.

$$\begin{array}{c} s^{CN}=\left|N_{i}\cap N_{j}\right|, \end{array}$$ (11)

where $N_{i}$ and $N_{j}$ represent the neighbors set of nodes *v*_*i*_ and *v*_*j*_, respectively.

(2) **RA**: [57] The basic idea of the RA is to evenly distribute one unit of resource to its neighbor nodes.

$$\begin{array}{c} s^{RA}=\sum_{z\in N_{i}\cap N_{j}}\frac{1}{k_{z}}, \end{array}$$ (12)

where the *k*_*z*_ represents the degree of node *z*.

(3) **Jaccard**: [58] Building upon the CN index, the impact of degree-corrected similarities of the nodes at either end of the link was taken into consideration.

$$\begin{array}{c} s^{Jaccard}=\frac{\left|N_{i}\cap N_{j}\right|}{\left|N_{i}\cup N_{j}\right|}. \end{array}$$ (13)

(4) **Node2vec**: [59] The optimization goal of Node2vec is to maximize the probability of observing neighboring nodes given a particular node.

$$\begin{array}{c} \underset{f}{\text{max}}\;\sum_{u\in V}\text{log}\;P\left(N_{I}\left(u\right)|f\left(u\right)\right), \end{array}$$ (14)

where *f*(*u*) is a learnable function that maps the node *u* to an embedding vector. *N*_*I*_(*u*) is the set of neighboring nodes of node *u* obtained through the sampling strategy *I*.

(5) **DeepWalk**: [60] learns the latent representation of nodes by node sequences, i.e,

$$\begin{array}{c} P(v_{i}|\left(v_{1},v_{2},\cdots ,v_{i-1}\right)). \end{array}$$ (15)

(6) **Metapath2vec**: [41] maximizes the local structure of different types of nodes,

$$\begin{array}{c} \text{arg}\;\underset{\theta }{\text{max}}\sum_{v\in V}\sum_{t\in T_{v}}\sum_{c_{t}\in N_{t}\left(v\right)}\text{log}\;p\left(c_{t}|v;\theta \right), \end{array}$$ (16)

where the *θ* is the parameter to be learned.

(7) **HAN**: [53] takes into account that different meta-paths have different weights on embedding learning.

$$\begin{array}{c} Z=\sum_{i=1}^{P}\beta_{\Phi_{i}}\cdot Z_{\Phi_{i}}, \end{array}$$ (17)

*Z* represents the embedding matrix, *P* is the number of meta-paths, $\beta_{\Phi_{i}}$ is the weight of the meta-path Φ_*i*_, and $Z_{\Phi_{i}}$ is the node embedding vector in terms of meta-path Φ_*i*_.

(8) **GAT**: [61] automatically learns the contribution levels of neighboring nodes when fusing features.

$$\begin{array}{c} h_{i}^{l+1}=\sum_{j\in N_{i}}\alpha_{ij}W^{\left(l\right)}h_{j}^{l}. \end{array}$$ (18)

(9) **RGCN**: [62] performs embedding learning separately for different types of links and then aggregates information from neighboring nodes.

$$\begin{array}{c} h_{i}^{\left(l+1\right)}=\sigma (\sum_{r\in R}\sum_{v_{j}\in N_{i}^{r}}\frac{1}{c_{i,r}}W_{r}^{\left(l\right)}h_{j}^{\left(l\right)}+W_{0}^{\left(l\right)}h_{i}^{\left(l\right)}), \end{array}$$ (19)

$N_{i}^{r}$ represents the neighbor nodes of *v*_*i*_ that the link type is *r* between them, R is a link type set in a heterogeneous network; *c*_*i*,*r*_ is a regularization constant, the usual value is $\left|N_{i}^{r}\right|$; $W_{r}^{\left(l\right)}$ is a linear transformation function.

(10) **GraphSage**: [63] aggregates feature information from both neighboring nodes and the node itself.

$$\begin{array}{c} h_{i}^{\left(l+1\right)}=\sigma (W\cdot concat(h_{i}^{\left(l\right)},aggregate\left\{h_{j}^{\left(l\right)},\forall v_{j}\in N_{i}\right\})). \end{array}$$ (20)

### Evaluation metrics

Recent studies have shown that traditional evaluation metrics for link prediction algorithms can sometimes misrepresent their true predictive performance [64, 65]. Inspired by the literature [66, 67], This paper selects threshold-free evaluation metrics AUC and Precision, where we set *L* equal to the number of edges in the test set when calculating Precision. In terms of the discriminative ability of evaluation indicators, AUC indicator has the strongest discriminative ability; For Precision, at this point, the Precision and Recall metrics are equal, making Precision calculations simpler and faster, and providing an additional evaluation perspective. On the other hand, Precision has been widely used in link prediction research and can still serve as a reference standard when comparing new and old algorithms or evaluation methods. Therefore, it is reasonable for this paper to choose AUC and Precision as indicators for link prediction evaluation.

In detail, the AUC is defined as follows,

$$\begin{array}{c} AUC=\frac{n^{\prime }+0.5n^{\prime \prime }}{n}, \end{array}$$ (21)

where *n*′ is the number of times that the edge score in the test set is greater than the non-existing edge score, and *n*″ is the number of times that those equal to each other, the *n* is the number of times that independent comparisons.

Precision only cares about whether some important links are accurately predicted, especially if the prediction can guide specific scientific experiments. Precision is defined as the proportion of the top *L* predicted links that are accurately predicted. Specifically, if *m* out of the top *L* predicted links are accurately predicted, the precision is given by

$$\begin{array}{c} Precision=\frac{m}{L}. \end{array}$$ (22)

In this paper, we set the *L* as number of test links. In other words, the Precsion measures the performance of the link prediction algorithm in all missing links.

### Results for SW-Metapath2vec and benchmark algorithms

The experiments of the SW-Metapath2vec algorithm consist of four main steps. Firstly, the meta-path traces are generated. Secondly, each trace is used as input for weighted embedding learning. Thirdly, link prediction is conducted using the embedding vectors. Lastly, the sensitivity analysis of the hyper-parameters is explored.

#### Step 1. Generate meta-path traces

**Fig 5:**
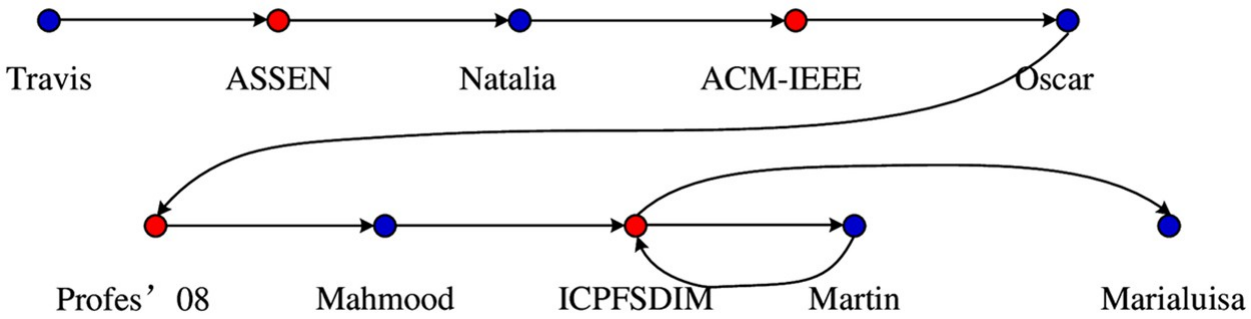
A meta-path trace for “APTPVPA” with author Travis as the initial node.

For each heterogeneous network, the meta-path traces are generated according to the meta-path shown in Table 2. For example, when we study the possible links in the DBLP network, we provide the “APTPVPA” for the meta-path P as shown in Fig 1. Taking the initial node “Travis A. Bennett” as an example, its meta-path trace is shown in Fig 5. To highlight the semantic relationship of the meta-path, Fig 5 omits the “paper” (P) nodes and the “term” (T) nodes, but it is still a meta-path trace of “APTPVPA”. In Fig 5, The blue nodes represent the authors, red nodes represent the name of the journal or conference. The first node is the author “Travis” (A), the second node is the journal “Assen” (V), and the third node is the author “Natalia” (A). Thus, an “APTPVPA” meta-path trace is generated, and the rest may be deduced by analogy.

#### Step 2. SW-Metapath2vec embedding learning

After all the meta-path traces are generated, they are used as input for the SW-Metapath2vec embedding algorithm, which learns the potential representation of network nodes. As shown in the flowchart in Fig 3, the SW-Metapath2vec embedding algorithm is primarily implemented using the deep learning framework PyTorch 1.4.0.

#### Step 3. Link prediction

In this experiment, we compare the SW-Metapath2vec algorithm with benchmark algorithms that have been introduced in Section: baseline algorithms. Notes that when we conduct experiments on ER networks and WS networks, we only generate the given length random walk sequence.

First, we conducted link prediction comparative experiments on two synthetic networks. The AUC values of each algorithm are shown in Table 3. The SW-Metapath2vec exhibits outstanding performance on synthetic networks. meanwhile, The embedding algorithms outperform the traditional similarity index.

**Table 3:** The AUC of the SW-Metapath2vec and the networks-based and embedding baselines (The test ratio is 0.3).

Networks	CN	RA	Jaccard	Node2Vec	Deepwalk	Metapath2vec	Ours
ER	0.5025	0.5336	0.5264	0.6241	0.6005	0.6305	**0.6430**
WS	0.7366	0.7435	0.7364	0.7925	0.7826	0.8219	**0.8230**

Graph neural networks have been proven to have strong capabilities in the field of link prediction. In order to compare the link prediction performance of SW-Metapath2vec and graph neural networks, we will focus on the comparison between them. we conducted detailed link prediction experiments on different types of link in the heterogeneous complex networks, as shown in Tables 4–6. They show the Precision of the SW-Metapath2vec algorithm and four benchmark graph neural networks on the ACM, DBLP, and Last.fm datasets, respectively. As seen in Table 4, on the ACM heterogeneous complex network, the SW-Metapath2vec algorithm demonstrates satisfactory predictive performance, particularly for the “paper-author” link type, consistently outperforming the baseline methods.

**Table 4:** The precision of the SW-Metapath2vec and the benchmark algorithms on the ACM network.

Edges type	Test ratio	HAN	GAT	RGCN	GraphSage	Ours
paper-field	30%	0.7891	0.8233	**0.8926**	0.8289	0.8522*
	40%	0.8279	0.8191	**0.8849**	0.8285	0.8443*
	60%	0.7869	0.8149	**0.8847**	0.8272	0.8662*
paper-author	30%	0.7674	0.7861*	0.7844	0.7813	**0.8484**
	40%	0.7639	0.7865*	0.7826	0.7796	**0.8582**
	60%	0.7444	0.7854*	0.7825	0.7694	**0.8503**

Table 5 presents the precision of various link prediction algorithms applied to the DBLP heterogeneous complex network. Overall, the SW-Metapath2vec algorithm demonstrates commendable predictive performance, particularly in predicting “author-paper” links, where it achieves the highest precision. However, for “paper-venue” and “paper-term” link predictions, its precision falls short compared to the HAN and RGCN graph neural network algorithms. This difference can be attributed to the relative sparsity of “paper-venue” and “paper-term” links within the DBLP network, which leaves many nodes disconnected through these link types. As a result, the SW-Metapath2vec algorithm did not effectively learn from and train the nodes associated with “paper-venue” and “paper-term” links.

**Table 5:** The precision of the SW-Metapath2vec and the benchmark algorithms on the DBLP network.

Edges type	Test ratio	HAN	GAT	RGCN	GraphSage	Ours
author-paper	30%	0.7173	0.7348	0.7307	0.7457*	**0.8484**
	40%	0.7177	0.7335	0.7329	0.7467*	**0.8539**
	60%	0.7146	0.7335	0.7294	0.7445*	**0.8511**
paper-venue	30%	**0.8990**	0.7450	0.8390	0.8548	0.8758*
	40%	**0.9223**	0.7125	0.7561	0.8508	0.8708*
	60%	**0.9119**	0.5896	0.7811	0.8468	0.8658*
paper-term	30%	0.8459	0.6125	**0.8839**	0.8182	0.8552*
	40%	0.8544*	0.5815	**0.8842**	0.8208	0.8492
	60%	0.7349	0.5916	**0.8861**	0.8161	**0.8502**

Table 6 presents the link prediction precision of the proposed SW-Metapath2vec algorithm compared to benchmark algorithms on the Last.fm dataset. As shown, SW-Metapath2vec outperforms the benchmark graph neural network algorithms in predicting “user-artist” and “user-user” links. However, for “artist-tag” link predictions, SW-Metapath2vec performs worse than the HAN, GAT, and RGCN algorithms. This discrepancy is primarily due to the relative sparsity of “artist-tag” links, which hinders the SW-Metapath2vec algorithm’s ability to effectively train and learn from the nodes associated with these links.

**Table 6:** The precision of the SW-Metapath2vec and the benchmark algorithms on the Last.fm network.

Edges type	Test ratio	HAN	GAT	RGCN	GraphSage	Ours
artist-tag	30%	0.8885*	0.8278	**0.8938**	0.8357	0.8594
	40%	0.8885*	0.8787	**0.8895**	0.8351	0.8501
	60%	**0.9010**	0.7844	0.8915*	0.8328	0.8536
user-artist	30%	0.7272	0.8324	0.8251	0.7821	**0.8482**
	40%	0.7142	0.8318*	0.8261	0.7815	**0.8640**
	60%	0.6697	0.8331*	0.8261	0.7785	**0.8521**
user-user	30%	0.7849	**0.8579**	0.8138	0.8195	0.8461*
	40%	0.7839	0.8248*	0.8111	0.8101	**0.8501**
	60%	0.7754	0.8105*	0.8045	0.7995	**0.8551**

Overall, the proposed SW-Metapath2vec link prediction algorithm demonstrates notable advantages, especially for networks with denser links. However, when a particular type of link is relatively sparse in the network, the imbalance in node sampling can prevent some nodes from being fully trained and learned. This limitation leads to a decrease in link prediction performance for sparse link types.

#### Step 4. Hyper-parameters sensitivity analysis

**Fig 6:**
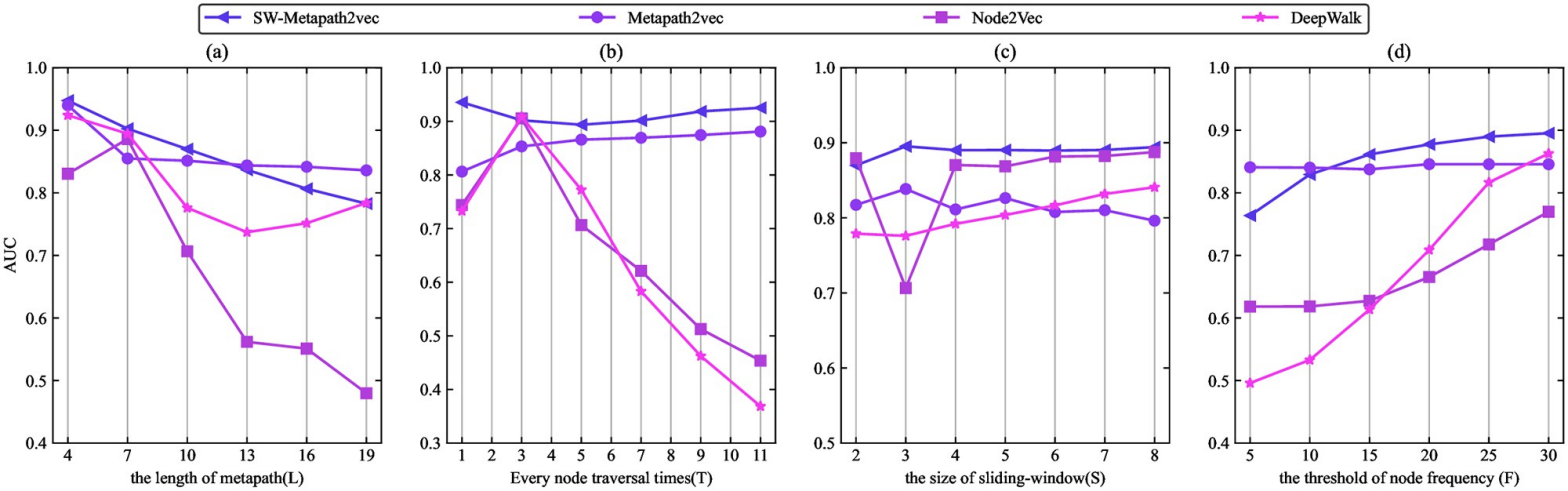
The AUC of SW-Metapath2vec and benchmark algorithms on DBLP with various hyper-parameters.

**Fig 7:**
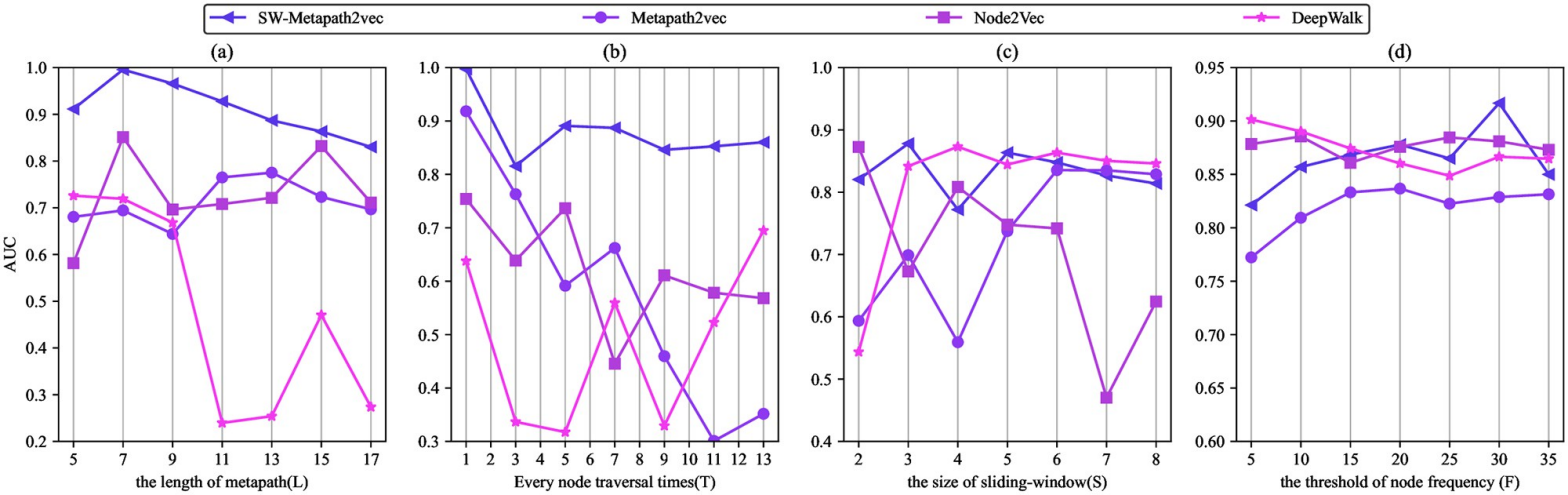
The AUC of SW-Metapath2vec and benchmark algorithms on Last.fm with various hyper-parameters.

To investigate the sensitivity of the hyper-parameters in the proposed SW-Metapath2vec link prediction algorithm in this paper, the sensitivity analysis is conducted on DBLP and Last.fm datasets according to the four main hyper-parameters used in the numerical experiment, which contains meta-path trace or path length *L*, node traversal times *N*, node frequency threshold *F*, and sliding window size *S*. The controls of the scale of “context” for each embedding learning, while *S* becoming greater, the “context” information is richer. Let benchmark hyper-parameters quadruples be (11, 5, 3, 10). We can change one of the parameters in each experiment. Figs 6 and 7 show the trend of the AUC of SW-Metapath2vec and other baseline algorithms concerning *L*, *N*, *F* and *S*, respectively.

As shown in Figs 6 and 7, the performance of the SW-Metapath2vec algorithm proposed in this paper surpasses that of the benchmark algorithms. With the increase in the length of the meta-path *L*, the AUC of SW-Metapath2vec decreases slowly, but it remains superior to the benchmark algorithms. Notably, a meta-path length of *L* = 5 is identified as a relatively optimal value. Increasing the number of node traversals enhances the stability of SW-Metapath2vec compared to other benchmark algorithms. In Fig 6(c), varying the size of the sliding window does not significantly affect the AUC of any of the algorithms. However, the AUC of SW-Metapath2vec declines when *F* = 30. Figs 6(c) and 7(c) also demonstrate that as the sliding window size increases, the AUC of the SW-Metapath2vec algorithm remains more stable and higher than that of the benchmark algorithms. From Figs 6(d) and 7(d), it is evident that appropriately increasing the threshold of node frequency helps improve the link prediction AUC for each algorithm by eliminating noise nodes, particularly in the large DBLP dataset.

### Robustness of the SW-Metapath2vec algorithm

**Fig 8:**
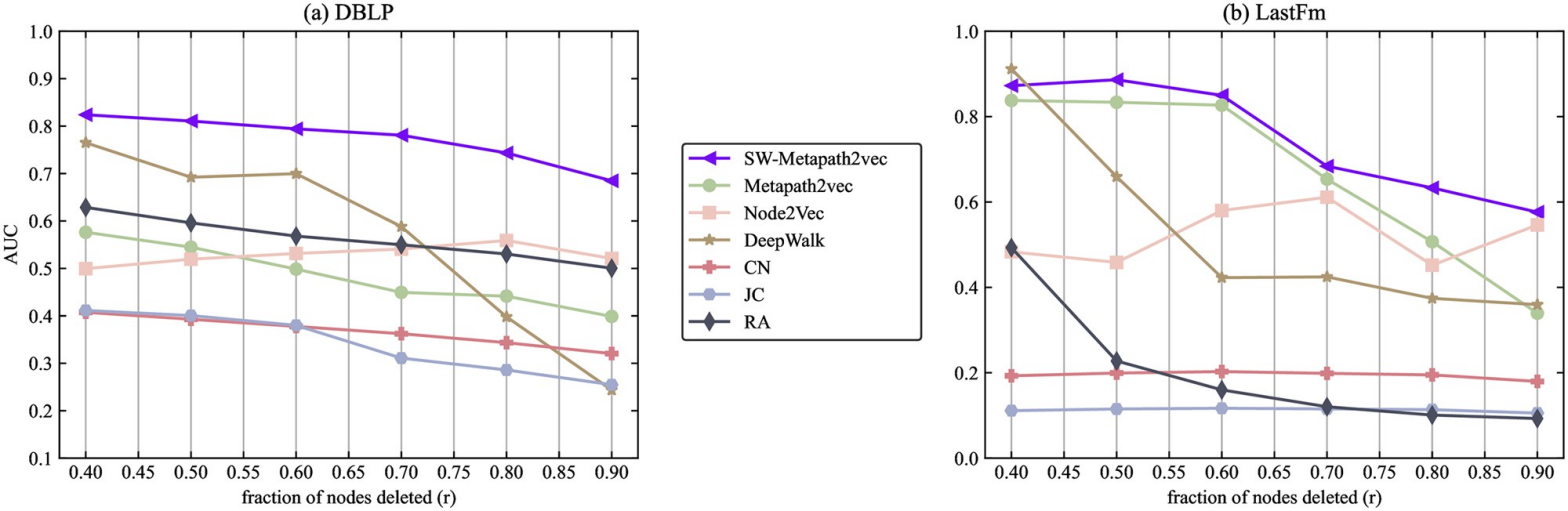
The AUC of SW-Metapath2vec and benchmark algorithms with a different fraction of nodes deleted.

In this section, to illustrate the robustness of the SW-Metapath2vec algorithm, we will delete a certain proportion of nodes from the DBLP and Last.fm datasets. And Fig 8 displays the AUC of all the algorithms versus the fraction of nodes deleted.

From Fig 8, it is evident that the robustness of the SW-Metapath2vec algorithm is stronger than that of other baseline algorithms. In the “paper-author” network, the SW-Metapath2vec algorithm demonstrates the best robustness. As the ratio of deleted nodes ranges from 0.4 to 0.9, the AUC of the SW-Metapath2vec algorithm remains around 0.8, outperforming other algorithms. In the “user-artist” network, SW-Metapath2vec experiences slight fluctuations but still maintains greater robustness compared to the benchmark algorithms.

## Discussion

In the 21st century, the natural environment, artificial environment, and human society can be described as large and diverse complex networks, specifically heterogeneous complex networks. Heterogeneous networks differ significantly from homogeneous networks due to their vast and diverse nodes and the complex links among them. As a result, many universal concepts applicable to homogeneous complex networks are no longer valid for heterogeneous networks. Traditional embedding learning methods have been crucial for link prediction in homogeneous complex networks, focusing on maximizing the likelihood of the local structure.

In heterogeneous networks, not all local links carry the same importance. To address this issue, this paper introduces a novel embedding learning objective function that assigns weight values to different local structures within heterogeneous networks and proposes a heterogeneous network link prediction algorithm called SW-Metapath2vec. The SW-Metapath2vec algorithm generates an embedding vector for each node in the heterogeneous network. It then calculates the similarity between any two nodes using the cosine similarity index, which indicates the potential for links between nodes. Experimental results on both real and synthetic datasets demonstrate that the proposed SW-Metapath2vec algorithm outperforms other benchmark algorithms in terms of performance and robustness. Specifically, the SW-Metapath2vec algorithm remains highly effective even when a large proportion of nodes are removed from the network.
